# Growth performance, intestinal morphology, and serum biochemistry of European quail (*Coturnix coturnix*) fed agro-industrial fruit residue extracts as alternatives to antibiotic growth promoters

**DOI:** 10.1016/j.psj.2026.107492

**Published:** 2026-07-26

**Authors:** Humberto de Araújo Brito Filho, Adiel Vieira de Lima, Cícero Jorge de Medeiros, Apolônio Gomes Ribeiro, Edijanio Galdino da Silva, Carlos Henriquedo Nascimento, Aline Beatriz Rodrigues, Paloma Eduarda Lopes de Souza, Ricardo Romão Guerra, Amanda Itoh de Medeiros, Thácyla Beatriz Duarte Correia, Matheus Ramalho de Lima, Leonardo Augusto Fonseca Pascoal, Lucas Rannier Ribeiro Antonino Carvalho, Fernando Guilherme Perazzo Costa

**Affiliations:** a Department of Animal Science, Federal University of Paraiba, Highway PB-079, Areia – PB, 58397-000, Brazil; b Department of Veterinary Sciences, Federal University of Paraíba, Areia, PB, Brazil; c Animal Science Department, Universidade Federal Rural do Semi-Árido, Mossoró, RN, Brazil; d Animal Science Department, Universidade Federal da Paraíba, Bananerias-PB, Brazil; e Department of Physiology and Pharmacology, Karolinska Institutet, Biomedicum 5B, Solnavägen 9, Stockholm, S-171 77, Sweden

**Keywords:** Natural additives, Fruit residues, Intestinal health, Performance, Quail

## Abstract

This study evaluated the effects of dietary supplementation with agro-industrial fruit residue extracts as alternatives to antibiotic growth promoters on performance, intestinal histomorphometry, and serum biochemical parameters of European quail (*Coturnix coturnix*). A total of 250 one-day-old birds were distributed in a completely randomized design with five treatments: positive control (bacitracin zinc, 500 g/t), negative control, and diets supplemented with pineapple (*Ananas comosus*) extract (900 ppm), acerola (*Malpighia emarginata*) extract (1,600 ppm), or cajá (*Spondias mombin*) extract (900 ppm) for 35 days. Data were subjected to analysis of variance (ANOVA), and means were compared using Tukey’s test at a 5% significance level. From 1 to 14 days, quail fed cajá extract showed lower weight gain compared with the negative control, with no differences for feed intake or feed conversion. From 15 to 35 days, feed intake was lower in birds receiving cajá compared with both controls. At 35 days, final body weight was lower in the cajá group, whereas pineapple and acerola treatments did not differ from the controls. In the ileum, villus height, villus area, and villus:crypt ratio were higher in birds fed pineapple and acerola extracts compared with cajá. Cajá extract resulted in lower villus height and villus:crypt ratio and higher crypt depth. In the duodenum and jejunum, fewer differences were observed among treatments. Serum biochemical parameters were affected by treatments. Birds fed cajá extract showed lower glucose, phosphorus, total protein, and albumin levels. Aspartate aminotransferase and alkaline phosphatase activities were higher in birds fed acerola extract. No differences were observed for triglycerides, creatinine, uric acid, alanine aminotransferase, or γ-glutamyl transferase. In conclusion, pineapple extract at 900 ppm and acerola extract at 1,600 ppm maintained performance and improved ileal morphology, whereas cajá extract at 900 ppm resulted in lower performance, altered intestinal morphology, and reduced serum biochemical parameters.

## Introduction

The use of antibiotic growth promoters in animal production has been established as an effective strategy to improve zootechnical performance, mainly through modulation of the intestinal microbiota and reduction of potentially pathogenic microorganisms (Santos, 2022). However, increasing concerns regarding antimicrobial resistance have led to restrictions on the non-therapeutic use of these compounds, since their continuous use is associated with the selection of resistant bacteria with potential impacts on human health (Tang et al., 2019; WHO, 2017). In this context, the search for natural alternatives capable of maintaining productive performance without compromising biosecurity has become a priority in poultry nutrition.

Among the proposed alternatives, phytogenic additives stand out, consisting of plant secondary metabolites with antimicrobial, antioxidant, and anti-inflammatory properties (Alves, 2023). These compounds, such as phenolics, flavonoids, and terpenes, play an ecological defense role in plants and may positively modulate the intestinal microbiota and the physiological status of animals (Taiz and Zeiger, 2004; Verruck et al., 2018). However, the results are still inconsistent, varying according to animal species, plant matrix, and administered dose.

Agro-industrial residues from tropical fruits represent a promising source of these bioactive compounds. In pineapple (*Ananas comosus*), polyphenols and bromelain stand out, being associated with antioxidant and anti-inflammatory activities (Oliveira et al., 2018; Pacheco et al., 2022). Cajá (*Spondias mombin*) presents a high content of phenolic compounds and flavonoids, with antioxidant and antimicrobial potential described in the literature (Silva et al., 2012; Neves et al., 2021). Acerola (*Malpighia emarginata*) is recognized for its high vitamin C content and phenolic compounds with significant antioxidant capacity (Marques et al., 2017; Ferreira et al., 2021). In addition to the functional potential, the use of these residues adds value to the production chain and contributes to agro-industrial sustainability.

In meat-type quail production, especially with European quail (*Coturnix coturnix*), there is still a lack of scientific information regarding the use of plant extracts as substitutes for antibiotic growth promoters, despite the increasing professionalization of the sector. Considering the need for viable alternatives and the variability of responses observed in the literature, it is essential to evaluate the effect of strategic inclusion levels of these extracts.

Therefore, the present study aimed to evaluate the zootechnical performance, intestinal integrity, and serum biochemical profile of European quail subjected to the replacement of antibiotic growth promoters with high doses of ethanolic extracts from agro-industrial residues of pineapple, cajá, and acerola, comparing them with positive and negative control treatments.

## Materials and methods

This study was conducted at the Poultry Research Laboratory of the Federal Rural University of the Semi-Arid (UFERSA), located in the municipality of Mossoró, Rio Grande do Norte, Brazil. All protocols and procedures followed animal welfare guidelines and were approved by the Ethics Committee on the Use of Animals (CEUA) of UFERSA (23/2025).

### Animal husbandry and management

A total of 250 European quail (*Coturnix coturnix*) were used, acquired from a commercial hatchery at 1 day of age. The birds were housed in galvanized wire cages measuring 70 × 50 × 30 cm. The lighting program adopted was 24 h of light (natural + artificial). The experiment lasted 35 days, divided into two phases: 1 to 14 and 15 to 35 days. The quail groups were mixed, with no separation by sex.

Water and feed were provided *ad libitum* throughout the rearing period. From 1 to 14 days, water was supplied using chick drinkers and feed in plate-type feeders; from 15 to 35 days, feed was provided in trough-type feeders and water in cup drinkers.

### Experimental diets and design

The birds were distributed into 5 treatments, arranged in a completely randomized design, with 5 replicates each, containing 10 birds per experimental unit. Before allocation to the experimental units, all birds were individually weighed to ensure uniform distribution among treatments and replicates.

The experimental treatments consisted of a positive control, with feed supplemented with an antibiotic (zinc bacitracin, 500 g/ton), and a negative control, without antibiotic. In addition, different supplementations were tested in the basal diet of the negative control: two treatments received 900 ppm/ton, one with agro-industrial pineapple residue extract and the other with agro-industrial cajá residue extract; the treatment with agro-industrial acerola residue extract received a dosage of 1,600 ppm/ton. All the aforementioned additives were added on top of the diet presented in Table 1.

**Table 1:** Proximate composition and calculated nutritional value of the diet.

Ingredients	**1 to 14 days**	**15 to 35 days**	Nutrients	**1 to 14 days**	**15 to 35 days**
Corn	46.780	52.113	Crude protein, %	25.00	23.00
Soybean meal	45.869	40.654	Metabolizable energy, kcal/kg	2966	3112
Soybean oil	3.365	4.378	Calcium, %	0.871	0.705
Limestone	1.021	0.758	Available phosphorus, %	0.363	0.318
Dicalcium phosphate	1.290	1.088	Sodium, %	0.170	0.150
Salt	0.393	0.344	Digestible lysine, %	1.401	1.178
L-lysine	0.121	0.022	Digestible methionine, %	0.819	0.542
DL-methionine	0.492	0.235	Digestible methionine + cystine, %	1.155	0.855
L-threonine	0.368	0.108	Digestible threonine, %	1.220	0.896
Mineral premix	0.150	0.150	Digestible tryptophan, %	0.299	0.271
Vitamin premix	0.150	0.150	Digestible valine, %	1.131	0.971
Total	100	100			

### Extraction of the extracts

**Fig. 1:**
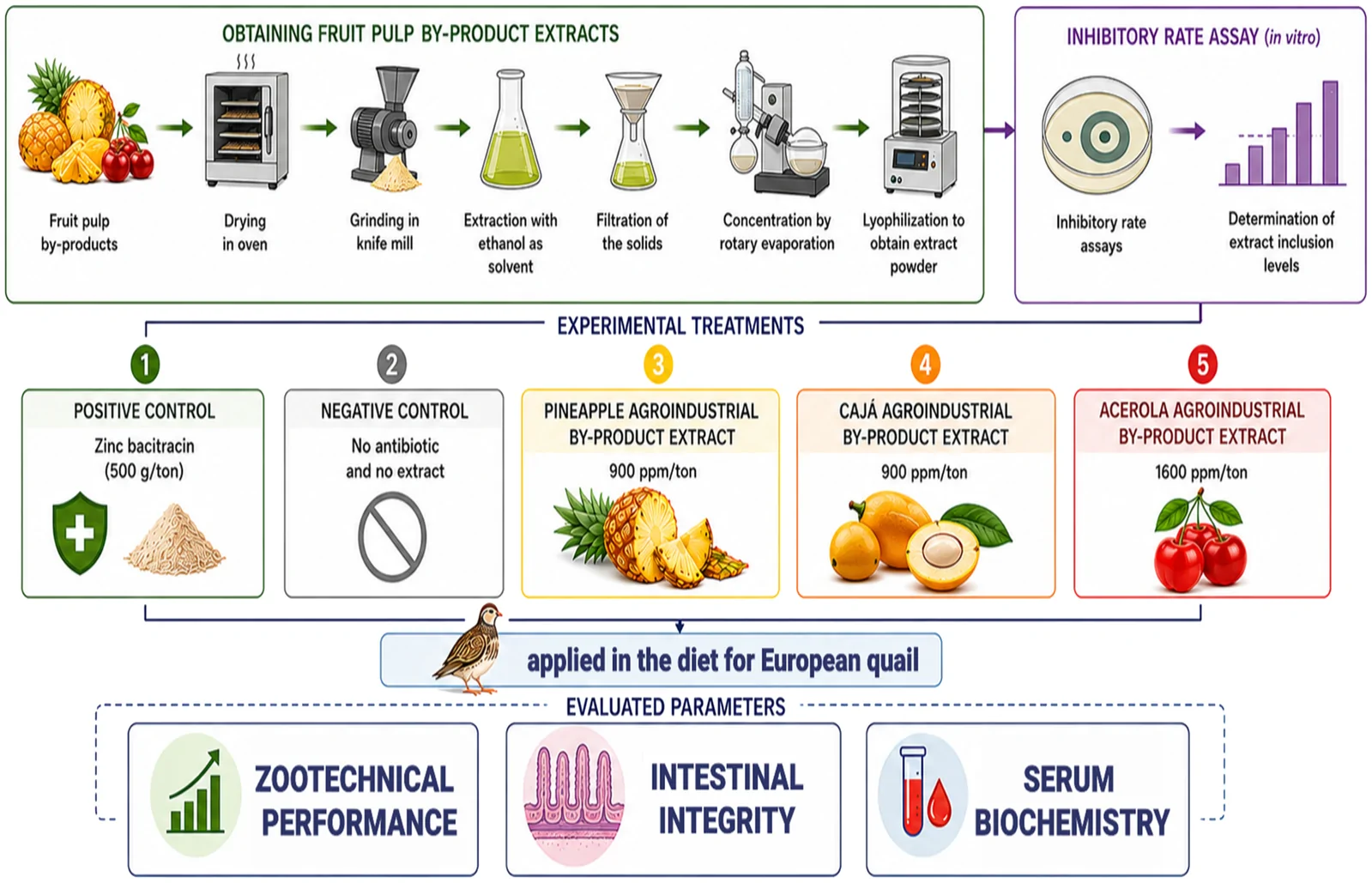
Schematic representation of the extraction process of agro-industrial fruit residues and their inclusion in experimental diets for European quails. Fruit pulp by-products were dried, ground, and subjected to ethanol extraction, followed by filtration, concentration by rotary evaporation, and lyophilization to obtain powdered extracts. The extracts were incorporated into the basal diet of the negative control (without antibiotic) at levels of 900 ppm/ton for pineapple (*Ananas comosus*) and cajá (*Spondias mombin*), and 1,600 ppm/ton for acerola (*Malpighia emarginata*).

Fruit pulp residues were obtained from a juice-producing industry (INTRAFRUT, João Pessoa, Paraíba, Brazil), oven-dried, and ground in a knife mill. The extracts were obtained with adaptations from the methodology of Caldas et al. (2018), using ethanol as the extraction solvent (Fig. 1). Briefly, 150 g of dried and ground residue was added to 1 L of a 50% ethanolic solution (500 mL of distilled water and 500 mL of ethanol). The mixture was maintained under agitation at 30°C for 1 h and subsequently vacuum-filtered. The solvent was removed using a rotary evaporator at 55°C to concentrate the extract.

Subsequently, 10% maltodextrin was added, according to Paiva et al. (2023), and the material was frozen for 24 h and lyophilized at −57°C for 72 h to obtain the dry extract. The resulting extracts were then subjected to inhibitory activity assays (MIC and MBC) to determine the appropriate inclusion levels. The range of concentrations tested (1.2 to 0.2 g/10 mL) was selected to encompass values capable of identifying the minimum effective inhibitory and bactericidal concentrations. The lowest bactericidal concentration (MBC) was subsequently used as the basis for defining inclusion levels in the following assays.

### Determination of minimum inhibitory concentration (mic) of microorganisms

The minimum inhibitory concentration (MIC) of the ethanolic extracts and their fractions was determined using a broth microdilution assay in 96-well microplates, adapted from the standard protocol M07-A9 of the Clinical and Laboratory Standards Institute (CLSI, 2012). For the assay, the standard inoculum was prepared in sterile NaCl solution (0.85% w/v) from fresh colonies of the selected bacteria grown on TSA agar (*E. coli*) or agar (*Salmonella* sp.), adjusted to an optical density equivalent to the 0.5 McFarland standard (0.08–0.13 at 625 nm).

Subsequently, the standard inoculum was diluted (1:100) to obtain a final inoculum of 10⁶ CFU/mL. Stock solutions of the ethanolic extracts and each fraction were prepared at concentrations of 1.2, 1.0, 0.8, 0.6, 0.4, and 0.2 g/10 mL in distilled water. From each stock solution, 180 µL was dispensed into the wells, and 20 µL of the final inoculum was added to each well.

The final volume in each well was 200 µL, resulting in a bacterial population of approximately 10⁵ CFU/mL. The following controls were included: culture medium control (20 µL of broth without inoculum) and positive control (180 µL of broth + 20 µL of *E. coli* or *Salmonella* inoculum). The plates were incubated at 37°C for 24 h.

The minimum bactericidal concentration (MBC) was determined from wells showing no visible bacterial growth. An aliquot of 100 µL from each well was plated onto Mueller–Hinton agar and incubated at 37°C for 24 h. The MBC was defined as the lowest concentration of ethanolic extract capable of causing bacterial death, as indicated by the absence of colony growth on agar plates. The lowest bactericidal concentration was used in subsequent assays (Fig. 1).

### Extraction and determination of phenolic compound profile

The extraction of phenolic compounds was performed using the Salting-Out Assisted Liquid–Liquid Extraction (SALLE) method, adapted from the methodology described by Shi et al. (2019). Initially, the fruit pulp was separated from the seeds and homogenized. Subsequently, 2.0 g of the sample was mixed with 2.0 mL of ultrapure water and 4.0 mL of acetonitrile, and the mixture was vortexed for 60 s. Then, 0.5 g of NaCl was added to promote phase separation, followed by vortex agitation for 3 min and centrifugation at 4,000 × g for 5 min.

After centrifugation, the supernatant phase was collected and subjected to evaporation in a rotary evaporator at 50°C. The dried extract was resuspended in 0.5 mL of 30% methanol and filtered through a 0.45 μm membrane prior to chromatographic analysis.

The identification and quantification of phenolic compounds were performed by reverse-phase high-performance liquid chromatography with a diode array detector (RP-HPLC/DAD), and the results are presented in Table 2.

**Table 2:** Identification and quantification of phenolic compounds in agro-industrial residue extracts.

**Compound**	**Pineapple (mg/L)**	**Acerola (mg/L)**	**Cajá (mg/L)**
3,4-Dihydroxybenzoic acid	—	18.55925	—
4-Hydroxybenzoic acid	6.35651	—	10.94776
Caffeic acid	—	1.50098	—
Chlorogenic acid	1.33205	—	—
Ferulic acid	2.47221	1.47039	2.63013
p-Coumaric acid	—	4.11536	—
Syringic acid	—	—	2.01033
trans-Cinnamic acid	—	2.31464	2.16677
Vanillic acid	—	4.17360	4.10908
Catechin	—	4.84563	8.74659
Epicatechin	—	4.71069	3.04086
Epicatechin gallate	12.58403	—	2.05084
Epigallocatechin gallate (EGCG)	10.73736	2.70225	7.70517
Hesperidin	—	69.35811	16.57821
Isorhamnetin	9.28068	—	—
Kaempferol-3-glucoside	—	41.37944	—
Naringenin	6.88000	3.99458	—
Naringin	4.43612	7.76262	2.17334
Pelargonidin-3-glucoside	—	8.84734	—
Procyanidin A2	—	1.78853	—
Procyanidin B1	—	—	14.98304
Procyanidin B2	1.65284	—	—
Quercetin (hydrate)	—	5.55683	3.73226
Quercetin-3-glucoside	—	23.53361	81.22657
Rutin	—	20.34259	—
trans-Resveratrol	2.59931	2.25527	—
Vanillin	—	—	4.27159
**Total General**	**58.33111**	**229.21171**	**166.37254**

### Zootechnical performance

The variables evaluated for zootechnical performance were feed intake (FI, g/bird), feed conversion ratio (FCR, g/g), and body weight gain (BWG, g/bird), with measurements determined at the end of the phases from 1 to 14 and 15 to 35 days.

For the evaluation of feed intake and feed conversion ratio, feed and birds were weighed at the beginning and at the end of each experimental phase. Feed intake was calculated as the difference between the amount of feed provided and the leftovers in each phase. Body weight gain was obtained as the difference between final and initial body weight. Feed conversion ratio was calculated as the ratio between feed intake and live weight. Mortality was recorded in each phase and used to correct the performance data.

### Carcass yield

At the end of the experiment, all birds were slaughtered, and three birds per experimental unit were used to determine carcass yield percentage. After bleeding, scalding, and defeathering, the birds were eviscerated, and the carcasses (without head and feet) were weighed.

For carcass yield determination, the weight of the clean and eviscerated carcass (without head and feet) was considered in relation to the fasting live weight obtained prior to slaughter.

### Intestinal histomorphometry

Samples from the middle portion of the ileum were collected from 6 birds per treatment, fixed in 10% formalin, and subjected to routine histological processing, embedded in paraffin, sectioned at 5 µm thickness, and stained with PAS (periodic acid–Schiff).

Digital images were obtained using a camera coupled to an Olympus BX-40 light microscope and analyzed using Motic Image Plus 2.0 software. The variables evaluated were villus height (VH), villus width, villus area, crypt depth, and villus:crypt ratio. Villus height was measured from the apex to its basal region, corresponding to the surface of the crypt to its apex; crypt depth was measured from the villus–crypt junction to its base; and villus width was measured at the apex (AW) and at the base of the villus (BW). For each animal, 10 measurements were performed, totaling 60 measurements. Based on the results obtained for villus height and crypt depth, the villus:crypt ratio was calculated. Villus area was calculated according to the equation ((AW + BW)/(VH × 2)).

### Serum biochemistry

Blood samples from 6 birds per treatment were collected by jugular vein puncture using sterile needles (13 × 0.4 mm) and placed in tubes without anticoagulant. The samples were allowed to rest for 30 min and then centrifuged at 3,500 rpm for 10 min to obtain serum. The serum was transferred to Eppendorf microtubes and stored frozen until analysis.

Biochemical analyses were performed at the Poultry Laboratory of the Federal University of Paraíba. Commercial kits (Labtest Diagnóstica S.A.®, Lagoa Santa, Brazil) were used to determine glucose (GLU; Ref. 133), triglycerides (TG; Ref. 87), cholesterol (CHOL; Ref. 1082), uric acid (UA; Ref. 140), urea (U; Ref. 1013), creatinine (CRE; Ref. 1010), aspartate aminotransferase (AST; Ref. 1009), alanine aminotransferase (ALT; Ref. 1008), gamma-glutamyltransferase (GGT; Ref. 1058), alkaline phosphatase (ALP; Ref. 1011), total protein (TP; Ref. 1085), albumin (ALB; Ref. 1007), calcium (Ca; Ref. 1084), and phosphorus (P; Ref. 12-200). All analyses were conducted using an automatic biochemical analyzer (Model SX-260, Manufacturer: Sinnowa, São Paulo, Brazil).

### Statistical analysis

Statistical analysis was performed using SAS software and included comparison of means conducted by ANOVA, followed by Tukey’s test at 5% probability.

## Results

Zootechnical performance variables of quails subjected to the experimental treatments are presented in Table 3. From 1 to 14 days, treatments did not show significant differences for feed intake (P = 0.4577) and feed conversion ratio (P = 0.2580). Regarding weight gain, the absence of supplementation was 9.67% more efficient than supplementation with cajá extract (P = 0.0293), while the other treatments did not differ statistically from each other.

**Table 3:** Productive performance and carcass yield.

Extrato	1 to 14 days			15 to 35 days			1 to 35 days			FBW	CY
	FI	BWG	FCR	FI	BWG	FCR	FI	BWG	FCR		
PC	10.98	6.49^ab^	1.69	23.62^a^	6.61	3.58	18.56^a^	6.56^a^	2.83	171.94^ab^	70.53
NC	11.35	6.69^a^	1.7	23.72^a^	6.5	3.65	18.77^a^	6.58^a^	2.86	182.64^a^	70.56
PINEAPPLE	11.45	6.43^ab^	1.78	22.53^ab^	6.25	3.6	18.10^ab^	6.32^ab^	2.86	168.47^ab^	70.94
CAJÁ	10.93	6.10^b^	1.79	21.56^b^	6.05	3.57	17.31^b^	6.07^b^	2.85	166.34^b^	70.74
ACEROLA	11.19	6.53^ab^	1.72	22.46^ab^	6.05	3.72	17.95^ab^	6.24^ab^	2.88	177.00^ab^	71.4
SEM	0.5243	0.2636	0.0890	0.9318	0.2946	0.1728	0.5926	0.2095	0.0960	8.3174	2.5638
*P-value*	0.4577	0.0293	0.2580	0.0082	0.0199	0.6478	0.0080	0.0046	0.9550	0.0366	0.9824

FI = Feed intake (g/bird/day); BWG = Body weight gain (g/bird/day); FCR = Feed conversion ratio (g/g); FBW = Final body weight (g); CY = Carcass yield (%). PC = Positive control (zinc bacitracin, 500 g/ton); NC = Negative control. Different letters within the same column indicate differences by Tukey’s test.

From 15 to 35 days, only feed intake was affected (P = 0.0082), with the positive and negative control treatments resulting in intake 9.55% and 10.02% higher, respectively, than the treatment supplemented with cajá extract. Body weight gain also differed among treatments (P = 0.0199), whereas feed conversion ratio was not affected (P = 0.6478). The other supplementation alternatives were similar to each other and did not differ statistically from the control treatments or from the cajá extract treatment.

From 1 to 35 days, feed intake (P = 0.0080) and body weight gain (P = 0.0046) differed among treatments, while feed conversion ratio did not (P = 0.9550). For the differences observed in feed intake and weight gain, the positive and negative control treatments were superior to the treatment with ethanolic extract of cajá agro-industrial residue, while the other extract treatments were similar to each other and to the control treatments. Final body weight differed among treatments (P = 0.0366), with the negative control being 9.80% higher than the cajá extract treatment, whereas carcass yield was not affected (P = 0.9824).

**Fig. 2:**
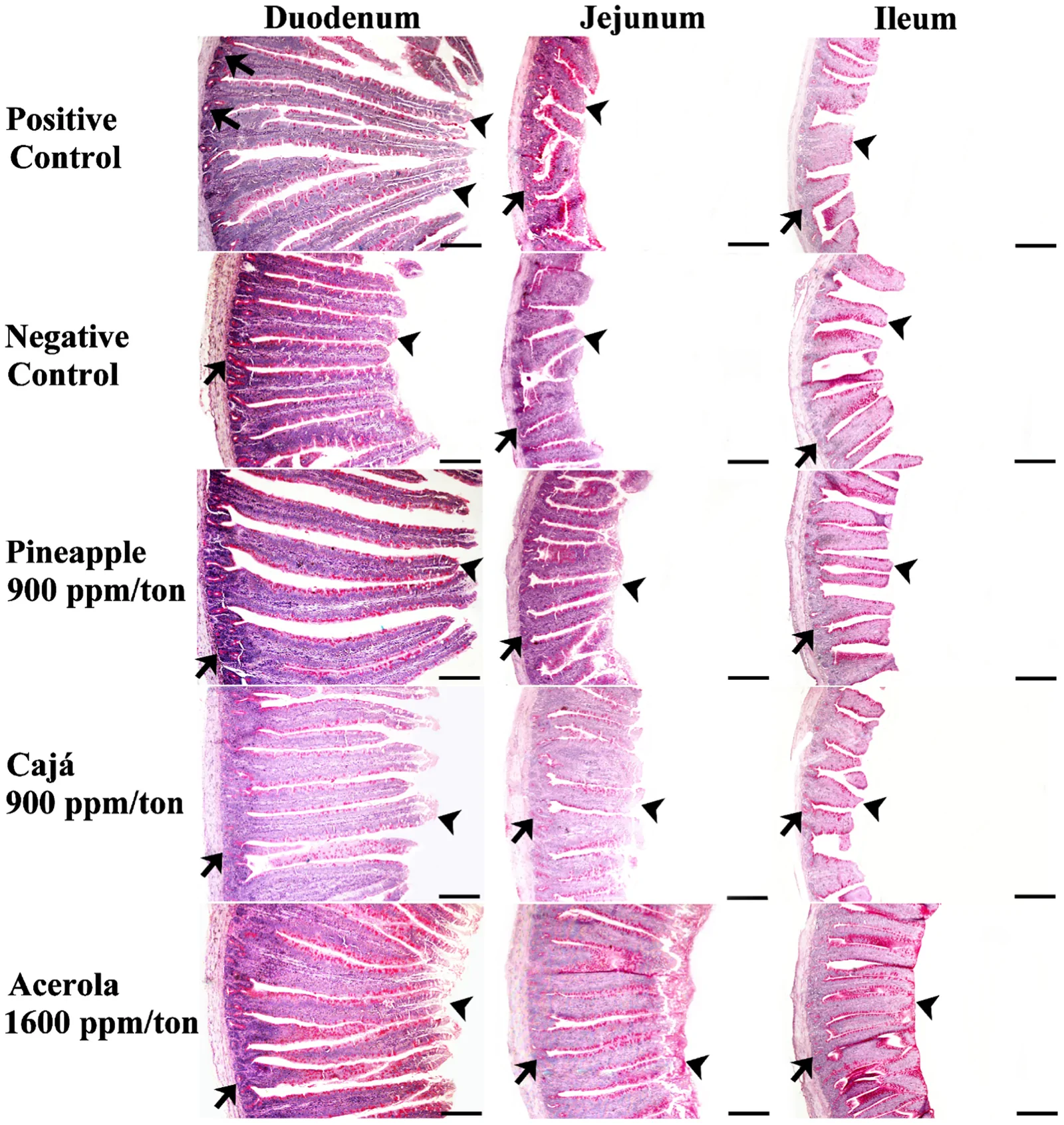
Photomicrographs of the duodenum, jejunum, and ileum of European quails subjected to different dietary treatments with agro-industrial extracts. Treatments: positive control (basal diet supplemented with zinc bacitracin, 500 g/ton); negative control (basal diet without antibiotic); Pineapple (negative control diet + pineapple residue extract, 900 ppm/ton); Cajá (negative control diet + cajá residue extract, 900 ppm/ton); Acerola (negative control diet + acerola residue extract, 1,600 ppm/ton). Intestinal morphology is shown according to segment and treatment. Crypts (arrows) and villi (arrowheads) are indicated. Periodic Acid–Schiff (PAS) staining with hematoxylin. Scale bars = 200 μm.

The intestinal integrity variables of the duodenum, jejunum, and ileum are presented in Table 4 and Fig. 2.

**Table 4:** Intestinal histomorphometry of the duodenum, jejunum, and ileum.

Treatments	PC	NC	Pineapple	Cajá	Acerola	SEM	*P-value*
	Duodenum						
Villus height	1052.57^ab^	929.96^c^	1092.43^a^	1016.33^b^	1020.87^b^	136.16	<0.001
Villus width	139.78^b^	123.99^c^	131.49^bc^	137.98b^c^	155.01^a^	32.48	<0.001
Villus area	147.830^a^	115.530^b^	145.591^a^	141.845^a^	157.683^a^	41.929	<0.001
Crypt depth	79.28	77.01	84.08	68.95	82.22	59.88	0.594
Villus:crypt ratio	17.18^a^	12.74^c^	13.34^c^	15.22^b^	12.96^c^	3.74	<0.001
	Jejunum						
Villus height	355.99^c^	397.94^bc^	420.86^ab^	477.25^a^	443.91^ab^	133.46	<0.001
Villus width	95.74^c^	105.31^bc^	103.45^bc^	117.33^a^	109.41^ab^	24.30	<0.001
Villus area	34.814^c^	42.161^bc^	43.715^bc^	58.004^a^	49.117^ab^	21.330	<0.001
Crypt depth	47.05^b^	45.46^b^	49.20^b^	55.46^a^	60.23^a^	12.30	<0.001
Villus:crypt ratio	7.82^bc^	9.07^a^	8.88^ab^	8.87^ab^	7.60^c^	2.69	0.001
	Ileum						
Villus height	297.22^ab^	279.44^bc^	310.11^a^	261.41^c^	319.96^a^	63.39	<0.001
Villus width	87.28	86.46	91.69	95.04	87.32	22.66	0.109
Villus area	25.525^abc^	23.486^c^	28.253^a^	24.721^bc^	27.818^ab^	7.715	0.001
Crypt depth	51.47^bc^	46.18^d^	48.62^cd^	57.69^a^	53.91^ab^	10.64	<0.001
Villus:crypt ratio	6.04^a^	6.33^a^	6.71^a^	4.74^b^	6.06^a^	1.84	<0.001

PC = Positive control (zinc bacitracin, 500 g/ton); NC = Negative control; SEM = Standard error of the mean. Different letters within the same row indicate differences by Tukey’s test.

In the duodenum, villus height was affected by treatments (P < 0.001). The absence of supplementation in the control treatment reduced villus height, being significantly lower than the other treatments. Supplementation with pineapple extract improved this variable, being statistically similar to the positive control, which in turn was similar to supplementation with cajá and acerola extracts.

For villus width, differences were observed among treatments (P < 0.001). Numerically, the positive control presented the lowest value and was statistically similar to supplementation with cajá and acerola extracts, while acerola extract showed the highest value.

Duodenal villus area differed among treatments (P < 0.001), with the negative control being inferior to the others, which were statistically similar. Crypt depth did not differ significantly among treatments (P = 0.594). For the villus:crypt ratio, significant differences were observed (P < 0.001), with the positive control presenting the highest value, followed by the cajá extract, whereas the remaining treatments were similar to each other.

In the jejunum, villus height differed among treatments (P < 0.001), with the highest value observed in the cajá extract, which did not differ statistically from acerola and pineapple extract treatments. Villus width also differed (P < 0.001), with the positive control showing lower values than cajá and acerola extract treatments.

Villus area was affected by treatments (P < 0.001), being higher in the cajá extract treatment, which was similar to acerola. Crypt depth differed among treatments (P < 0.001), with cajá and acerola extracts showing higher values compared to the others. The villus:crypt ratio also differed (P = 0.001), with the highest value observed in the control treatment, while acerola extract showed the lowest numerical value.

In the ileum, villus height was influenced by treatments (P < 0.001), with cajá extract showing the lowest value. Villus width did not differ among treatments (P = 0.109).

Ileal villus area differed among treatments (P = 0.001), being lower in the negative control and cajá extract treatments, while pineapple and acerola extracts resulted in higher values. Crypt depth was affected (P < 0.001), increasing with cajá extract supplementation. The villus:crypt ratio also differed (P < 0.001), with lower values observed in the cajá extract treatment, while no significant differences were observed among the remaining treatments.

The serum biochemical profile of European quails is presented in Table 5. Glucose was significantly affected among treatments (P = 0.011), with the value observed for cajá extract being lower, which was statistically similar only to the negative control and the pineapple extract treatment. Higher values were observed for acerola extract and the positive control, which differed from the cajá extract and were similar to the negative control and pineapple extract.

**Table 5:** Serum biochemistry of European quails supplemented with ethanolic extracts of agro-industrial fruit residues as substitutes for antibiotic growth promoters.

Tratamentos	CP	CN	Pineapple	Cajá	Acerola	SEM	*P-Value*	N
	Energy and intermediate metabolism							
GLU, mg/dl	320.67^a^	277.33^ab^	305.67^ab^	244.33^b^	331.17^a^	42.72	0.011	30
TG, mg/dl	946.00	309.00	888.5	165.88	273.33	980.81	0.504	30
CHOL, mg/dl	244.50^a^	200.00^ab^	220.83^ab^	117.82^b^	191.83^ab^	62.22	0.020	30
	Nitrogen compound metabolism							
UA, mg/dl	7.86	6.82	7.80	7.48	7.82	2.29	0.925	30
U, mg/dl	6.50	7.33	6.33	6.50	6.17	2.27	0.915	30
CRE, mg/dl	0.28	0.38	0.39	0.29	0.48	0.11	0.1043	30
	Hepatic function							
AST, U/L	248.83^ab^	163.50^b^	242.00^ab^	159.17^b^	265.17^a^	53.55	0.003	30
ALT, U/L	5.17	4.67	5.83	3.33	4.67	1.60	0.128	30
GGT, U/L	11.83	12.83	11.33	11.50	12.50	2.06	0.673	30
ALP, U/L	427.00^b^	328.67^b^	411.50^b^	660.67^ab^	1193.67^a^	421.84	0.010	30
TP, g/dl	2.70^a^	2.50^ab^	2.85^a^	1.58^b^	2.57^ab^	0.62	0.018	30
ALB, g/dl	1.48^a^	140^ab^	1.56^a^	0.92^b^	1.49^a^	0.28	0.005	30
	Mineral metabolism							
Ca, mg/dl	12.22	9.68	12.79	6.54	10.44	6.28	0.464	30
P, mg/dl	6.50^a^	4.43^ab^	5.75^ab^	3.34^b^	6.05^ab^	1.68	0.019	30

PC: Positive control; NC: Negative control; GLU: Glucose; TG: triglycerides; CHOL: cholesterol; UA: uric acid; U: urea; CRE: creatinine; AST: aspartate aminotransferase; ALT: alanine aminotransferase; GGT: gamma-glutamyl transferase; ALP: alkaline phosphatase; TP: total protein; ALB: albumin; Ca: calcium; P: phosphorus. SEM = Standard error of the mean; N = number of observations. Different letters within the same row indicate differences by Tukey’s test.

No significant differences were observed among treatments for triglycerides (P = 0.504). For cholesterol, a statistical difference was observed (P = 0.020), with the positive control showing a higher value than the cajá extract treatment; however, both were individually similar to the other treatments.

For variables related to nitrogen compound metabolism, no effect was observed among treatments (uric acid: P = 0.925; urea: P = 0.915; creatinine: P = 0.1043).

AST enzyme activity was affected by the treatments (P = 0.003), with a significant difference between the acerola extract treatment and the cajá extract and negative control treatments, while the acerola extract treatment was similar to the pineapple extract and positive control treatments. ALT and GGT enzymes did not show significant differences among treatments (P = 0.128 and P = 0.673, respectively).

Alkaline phosphatase was influenced by treatments (P = 0.010), being higher in the acerola extract treatment, which was statistically similar only to the cajá extract treatment.

Total protein differed among treatments (P = 0.018), with the cajá extract showing a lower value compared to the positive control, while remaining similar to the negative control and acerola extract. Albumin was also affected (P = 0.005), with the cajá extract presenting the lowest value and being statistically similar only to the negative control.

Serum calcium levels were not affected by treatments (P = 0.464). Serum phosphorus differed significantly among treatments (P = 0.019), with lower values observed in the cajá extract compared to the positive control.

## Discussion

A good zootechnical additive is characterized not by a “superpower,” but by its ability to remove barriers to performance. Zahed et al. (2025), for example, recommend the inclusion of 0.65% pistachio biochar in the diet of growing Japanese quails (*Coturnix japonica*) due to its significant improvements in growth and digestibility. However, the authors attribute this effect to the antimicrobial properties of biochar, which reduced the population of Escherichia coli and increased that of Lactobacillus, such that these microbial changes likely improved nutrient digestion and absorption. In addition, the adsorptive capacity of biochar may reduce antinutritional compounds and toxins.

Similarly, the action of antibiotic growth promoters, which are also referred to as performance enhancers - often leading to the assumption that they directly stimulate performance - is not to directly enhance animal performance, but rather to mitigate the effects of factors that may impair such performance.

Thus, in the absence of strong deleterious environmental factors that could be mitigated by the indirect action of antibiotic growth promoters, their use becomes ineffective. This hypothesis explains the behavior of the negative control treatment for performance variables, which did not impair feed intake or weight gain in any of the evaluated phases, nor final body weight, compared with the positive control treatment. It is likely that the sanitary challenge in the experimental environment was not sufficient to impose a relevant infectious pressure.

The aforementioned comparison between control treatments can be extended to the treatments with ethanolic extracts of pineapple and acerola agro-industrial residues. For performance variables, both also did not differ statistically from each other or from the control treatments.

The findings of the present study are consistent with the results reported by Diógenes (2017), who, when evaluating increasing levels of pyroligneous extract in a factorial scheme using new litter or used litter in meat-type quail production - where the latter condition is associated with higher sanitary pressure - demonstrated that the levels of pyroligneous extract supplementation did not influence weight gain and feed conversion of birds reared on new litter. However, for quails reared on used litter, a linear effect was observed for feed intake, weight gain, and feed conversion at inclusion levels of 0.0, 1.0, 1.5, 2.0, and 2.5%.

Ahmad et al. (2024), when discussing the use of ethanolic extract of Citrus sinensis in Japanese quails, state that its effectiveness on growth performance depends on several factors, such as the amount of extract used, its concentration and inclusion level, the physiological status of the birds, diet composition, and the active chemical compounds present. In this context, this statement aligns with the results found in the present study, in which the ethanolic extract of cajá agro-industrial residue reduced performance compared with the control treatments, especially the negative control. Although cajá residue contains bioactive compounds with antioxidant and antimicrobial potential, the biological response to plant extracts depends on their concentration, chemical profile, and interactions among compounds (Mahfuz et al., 2021; Rodrigues et al., 2022). In this context, the negative effects observed with cajá extract may be associated with the presence of phenolic compounds at concentrations that exceed the beneficial range, resulting in reduced nutrient utilization. Certain phenolic compounds, particularly tannins, may bind to dietary proteins and minerals, decrease digestive enzyme activity, and impair nutrient absorption (Cosme et al., 2025). Furthermore, excessive antimicrobial activity may alter the intestinal microbial balance, affecting gut functionality and nutrient availability. These mechanisms may explain the reduction in performance observed in quails supplemented with cajá extract.

Even when the presence of bioactive compounds in natural products is confirmed, as in the extracts evaluated in this study, the nutraceutical effect, when applied in animal diets, must positively impact performance to be considered a promising zootechnical additive, particularly when intended to replace antibiotic growth promoters.

However, in the organismal dynamics of commercial poultry, the nutraceutical effect is not necessarily associated with improved performance. For example, Dourado et al. (2025), when testing inclusion levels of 0, 1, and 2% buriti oil (*Mauritia flexuosa L.*) in broiler diets (from 1 to 21 days), concluded that it exerts a nutraceutical effect by improving diet utilization, increasing meat pigmentation, enhancing immune response, and stimulating collagen synthesis in the skin of birds; however, it impaired body weight, daily weight gain, and feed conversion.

Regarding the effects observed in intestinal integrity variables in the duodenum, jejunum, and ileum, there is greater agreement with the zootechnical performance results in the ileal segment, where a slightly more pronounced negative effect of the ethanolic extract of cajá agro-industrial residue was observed, especially in crypt depth and villus:crypt ratio, which were increased and decreased, respectively.

Nevertheless, the three extracts in the duodenal and jejunal segments, as well as the ethanolic extracts of pineapple and acerola residues in the ileum, were not detrimental. There were changes that favored them and others that were unfavorable compared with the control treatments.

The consistency of these results can be supported by the findings of Zhou et al. (2025). In a systematic review on the use of additives as substitutes for antibiotic growth promoters in quail production, when compiling the effects of plant extracts, the authors emphasize that these additives not only act by modulating the microbiome through antimicrobial activity, but also interact with quail physiology itself, such as stimulating digestive enzyme secretion, interfering with pancreatic enzyme activity and bile secretion, and promoting gastrointestinal motility. These direct effects on quails may have contributed to the histomorphological changes observed.

Additionally, Lima et al. (2025) state that the evaluation of blood biochemical parameters is an important tool for monitoring the physiological, nutritional, and sanitary status of commercial poultry.

For parameters related to energy and intermediate metabolism, a divergent pattern was observed between the cajá extract treatment and the positive control, which differed significantly for glucose and cholesterol.

Regarding glucose, the reduction observed in the extract treatment may be partially related to the effect described by Sevim (2026), who evaluated the inclusion of fermented leek powder (0, 2.5, 5.0, and 10.0 g/kg) in diets of growing male Japanese quails, where supplementation reduced serum glucose. This effect was attributed by the author to hypoglycemic properties and modulation of glucose metabolism associated with the presence of bioactive compounds, an attribution that can be extended to the present study. Additionally, the lower glycemia may be related to the lower feed intake observed in this treatment compared with the positive control.

Hassanabadi et al. (2026) attribute the removal of cholesterol from the bloodstream of Japanese quails to an increase in HDL-C, responsible for reverse cholesterol transport. However, the reduction in total cholesterol observed in the present study, especially in the cajá extract treatment compared with the positive control, should not be interpreted in isolation. The same authors, when evaluating the inclusion of mountain mint (Ziziphora clinopodioides Lam.) in the diet, observed a decrease in LDL-C and an increase in HDL-C, without significant changes in triglycerides and total cholesterol, suggesting that the effects of the additive are more related to modulation of lipoprotein distribution and composition than to a reduction in total lipid content.

In this sense, the results of the present study, characterized by the absence of significant differences in triglycerides and variation in total cholesterol levels among treatments, indicate a similar pattern, in which bioactive compounds act predominantly on lipid metabolism dynamics. Supporting this interpretation, Liu et al. (2025), using Artemisia selengensis Turcz leaves (100 mg/kg body weight/day) in Wistar rats, demonstrated that the treatment increased hepatic uptake, processing, and excretion of cholesterol, improving the lipid profile. This effect was associated with increased expression of genes such as CYP7A1, involved in the conversion of cholesterol into bile acids, and ABCA1, related to cholesterol transport and efflux.

Thus, it is evident that bioactive compounds present in phytotherapeutics do not act through simplistic mechanisms, such as direct reduction of plasma lipid levels, but rather through redistribution and modification of lipid composition rather than total lipid content. In addition, variations in dosage, duration of supplementation, bird genotype, and physiological stage may influence the lipid responses observed, contributing to divergences among different studies (Hassanabadi et al., 2026).

The absence of changes in nitrogen metabolites related to renal function indicates that the treatments employed did not compromise renal integrity. According to Lima et al. (2025), elevated uric acid concentrations may indicate renal or metabolic dysfunctions, whereas reduced levels may be associated with hypouricemia and renal or hepatic disorders. In birds, the urea cycle has limited participation in nitrogen excretion, being predominantly related to renal detoxification. Therefore, serum urea has low applicability in the direct diagnosis of renal disorders, although it may act as a sensitive indicator of hydration status and pre-renal alterations (Scope and Schwendenwein, 2020). Furthermore, increases in creatinine are uncommon and generally associated with severe renal failure (Capitelli and Crosta, 2013).

Khaleghipour et al. (2019), when evaluating supplementation of milk thistle (*Silybum marianum*) extract in diets of European quails from 7 to 35 days at levels of 0, 1,000, and 2,000 mg/kg, associated or not with aflatoxin (0 and 2.2 mg/kg), observed that aflatoxin alone reduced feed intake and weight gain, whereas feed conversion and daily weight gain were improved only at the level of 1,000 mg/kg as an isolated effect of milk thistle extract. In parallel, urea was not influenced by the tested additive; however, aflatoxin alone reduced creatinine and increased uric acid. These findings corroborate the results of the present study regarding performance changes promoted by the extract, which did not result in corresponding modifications in nitrogen metabolites related to renal function. On the other hand, the changes triggered by aflatoxin in performance and the resulting alterations in creatinine and uric acid levels may indicate that these parameters are more closely associated with severe specific damage to quail health.

Regarding hepatic functions, according to Zaefarian et al. (2019), the avian liver performs essential roles in synthesis, metabolism, excretion, and detoxification, being considered an important biochemical center of the organism. Different nutritional strategies and feeding techniques can contribute to maintaining liver function and overall bird health. Hepatic functions exceed 2,000 described roles in the relevant literature. In general, liver health corresponds to a healthy metabolism (Khaleghipour et al., 2019).

However, the interpretation of hepatic biochemical markers in birds should be carried out with caution, since enzymes such as AST and ALP present low tissue specificity and may also be influenced by muscle, intestinal metabolism, and body growth (Lumeij and Westerhof, 1987). Thus, isolated changes in these parameters do not always characterize severe hepatic impairment.

The increase in AST and ALP observed in birds receiving acerola extract may reflect metabolic modulation induced by the bioactive compounds present in this residue rather than pathological hepatic damage (Laurindo et al., 2024). Acerola is recognized as a source of phenolic compounds and vitamin C, which may influence oxidative metabolism, cellular turnover, and nutrient utilization. In addition, AST activity is not exclusively associated with hepatic tissue in birds and may also increase due to alterations in muscle metabolism or physiological adaptation (Maia et al., 2025). Similarly, ALP may be influenced by intestinal activity, mineral metabolism, and tissue growth processes. Therefore, the elevation of AST and ALP in the acerola treatment should be interpreted as a possible adaptive metabolic response, especially considering the absence of changes in more liver-specific markers, such as ALT and GGT.

Regarding the variables that remained unchanged among treatments, ALT, compared with AST, is more specific to the liver and, consequently, a more appropriate parameter to detect hepatic injury. Elevated levels of these serum enzymes indicate cellular leakage and loss of functional integrity of the hepatic cell membrane (AbouGabal et al., 2015). Additionally, the absence of changes in GGT suggests that the treatments did not promote relevant hepatobiliary impairment, since this enzyme is more related to alterations of the biliary epithelium and cholestatic processes in birds, although it presents limited diagnostic sensitivity when evaluated in isolation (Lumeij and Westerhof, 1987).

In the present study, a distinct response was observed among the evaluated extracts, indicating possible metabolic modulation dependent on the bioactive composition of each agro-industrial residue. While acerola extract promoted an increase in AST and ALP, cajá extract reduced total protein and serum albumin, suggesting extract-specific modulation of hepatic and metabolic dynamics. However, these alterations should not be interpreted as evidence of hepatic injury, considering the absence of changes in more specific markers and the physiological roles of these enzymes in birds.

Numerous dietary nutrients directly influence growth, bone maintenance, and egg quality, particularly calcium and phosphorus (Souza et al., 2018). Calcium is crucial for bone formation, muscle contraction, and acid–base balance, whereas phosphorus contributes to cellular metabolism, growth, and nerve impulse transmission (Oliveira et al., 2025).

According to Blaine et al. (2014), the individual homeostasis of these ions in the organism, as well as their relationship, is regulated by physiological components such as bone absorption and resorption, renal regulation, and gastrointestinal absorption, being mediated by hormones. Therefore, homeostatic mechanisms were sufficient to maintain serum calcium levels similar among treatments, without significant statistical differences. On the other hand, serum phosphorus was significantly altered, with cajá extract reducing its level compared with the positive control.

Vieites et al. (2011), when testing eight levels of dietary electrolyte balance in broilers, ranging from 0 to 350 mEq/kg from 1 to 42 days, found that this alteration did not result in significant differences in plasma calcium and phosphorus levels, reinforcing the efficient physiological regulation present in commercial poultry. In the same study, significant differences among treatments for phosphorus were observed only when crude protein content, evaluated in isolation, was increased from 20% to 23% during the 1 to 21-day phase, resulting in an increase from 6.52 to 6.78 mg/dL. This result corroborates the present study regarding changes in serum phosphorus due to dietary modifications, which likely induce physiological changes that homeostatic mechanisms described by Blaine et al. (2014) are unable to fully neutralize.

The reduction in serum phosphorus levels observed in the cajá extract treatment may be related to the antinutritional action of phenolic compounds, such as tannins, which reduce nutrient digestibility and form insoluble complexes with minerals in the gastrointestinal tract, impairing phosphorus availability and absorption (Singh, 2008; Choi and Kim, 2020).

## Conclusion

The present study demonstrated that ethanolic extracts of pineapple and acerola agro-industrial residues are potential substitutes for antibiotic growth promoters for European quails under the experimental conditions employed. In contrast, cajá extract is not recommended, as it impairs weight gain, compromises ileal histomorphometry, and negatively alters the serum biochemical profile. Future studies should focus on optimizing inclusion levels, evaluating combinations with other functional additives, and assessing their effects under different production conditions, aiming to further expand the applicability of these natural feed additives in poultry nutrition.

## Disclosures

The authors declare that they have no known competing financial or personal interest that could have impact the outcome of the study reported in this paper.

## Author Contribution

Humberto de Araújo Brito Filho led the experimental study and was responsible for its execution, including animal management, preparation and administration of the experimental diets, collection and organization of experimental data, statistical analysis, interpretation of the results, and preparation of the original manuscript. Adiel Vieira de Lima contributed to the execution of the experiment, animal management, collection of experimental data, interpretation of the results, statistical analysis, and preparation and revision of the manuscript. Cícero Jorge de Medeiros contributed to the execution of the experimental procedures, interpretation of the experimental results, and development and refinement of the procedures used for the preparation and administration of the fruit residue extracts. Apolônio Gomes Ribeiro contributed to the execution of the experiment and field activities, collection and analysis of experimental data, interpretation of the results, organization of the study for publication, and preparation and revision of the manuscript. Edijanio Galdino da Silva contributed to the execution of the experiment, collection and processing of experimental samples, and analysis and interpretation of the intestinal histological data. Carlos Henrique do Nascimento contributed to the execution of the experiment, including animal management, collection of experimental data and samples, organization and processing of the data, and literature investigation. Aline Beatriz Rodrigues contributed to the execution of the experiment, including animal management, collection of experimental data and samples, organization and processing of the data, and literature investigation. Paloma Eduarda Lopes de Souza contributed to the execution of the experiment, including animal management, collection of experimental data and samples, organization and processing of the data, and literature investigation. Ricardo Romão Guerra contributed to the development and application of the intestinal histological procedures, processing and analysis of histological samples, interpretation of histological findings, and preparation of the manuscript. Amanda Itoh de Medeiros contributed to the execution of the experiment, including animal management, collection of experimental data and samples, organization and processing of the data, and literature investigation. Thácyla Beatriz Duarte Correia contributed to the execution of the experiment, including animal management, collection of experimental data and samples, organization and processing of the data, and literature investigation. Matheus Ramalho de Lima contributed to the execution of the experiment, collection and organization of experimental data, literature investigation, interpretation of the results, and preparation of the manuscript. Leonardo Augusto Fonseca Pascoal contributed to the design and development of the methodology for analysis of the fruit residue extracts, refinement of the experimental procedures, interpretation of the results, and literature investigation. Lucas Rannier Ribeiro Antonino Carvalho contributed to the execution of the experiment, research and literature investigation, development and refinement of experimental methodologies, collection and processing of data, interpretation of the results, and preparation and revision of the manuscript. Fernando Guilherme Perazzo Costa supervised and coordinated the study, contributed to the experimental design and methodological development, participated in the execution and interpretation of the research, and contributed to the preparation and revision of the manuscript.
